# Endoplasmic Reticulum Stress Induced Proliferation Remains Intact in Aging Mouse β-Cells

**DOI:** 10.3389/fendo.2021.734079

**Published:** 2021-08-31

**Authors:** Jarin T. Snyder, Christine Darko, Rohit B. Sharma, Laura C. Alonso

**Affiliations:** ^1^Graduate School of Biomedical Sciences, UMass Medical School, Worcester, MA, United States; ^2^Division of Endocrinology, Diabetes and Metabolism and the Weill Center for Metabolic Health, Weill Cornell Medicine, New York, NY, United States

**Keywords:** pancreatic beta cells, proliferation, endoplasm Reticulum Stress, unfolded protein response, ATF6 (activating transcription factor 6), thapsigargin, aging/aging beta cells

## Abstract

Aging is associated with loss of proliferation of the insulin-secreting β-cell, a possible contributing factor to the increased prevalence of type 2 diabetes in the elderly. Our group previously discovered that moderate endoplasmic reticulum (ER) stress occurring during glucose exposure increases the adaptive β-cell proliferation response. Specifically, the ATF6α arm of the tripartite Unfolded Protein Response (UPR) promotes β-cell replication in glucose excess conditions. We hypothesized that β-cells from older mice have reduced proliferation due to aberrant UPR signaling or an impaired proliferative response to ER stress or ATF6α activation. To investigate, young and old mouse islet cells were exposed to high glucose with low-dose thapsigargin or activation of overexpressed ATF6α, and β-cell proliferation was quantified by BrdU incorporation. UPR pathway activation was compared by qPCR of target genes and semi-quantitative *Xbp1* splicing assay. Intriguingly, although old β-cells had reduced proliferation in high glucose compared to young β-cells, UPR activation and induction of proliferation in response to low-dose thapsigargin or ATF6α activation in high glucose were largely similar between young and old. These results suggest that loss of UPR-led adaptive proliferation does not explain the reduced cell cycle entry in old β-cells, and raise the exciting possibility that future therapies that engage adaptive UPR could increase β-cell number through proliferation even in older individuals.

## Introduction

Type 2 diabetes is an age-associated chronic disease, with a peak incidence of 25% in the US population aged ≥65 (1). Lifestyle interventions targeting people with prediabetes delay onset and reduce incidence of disease (2), but most people pass through the prediabetes phase without being diagnosed, and multiple barriers limit implementation of successful lifestyle changes. Treatments targeting aging as a risk factor for diabetes are lacking. As an epidemic of obesity coincides with population aging in the coming decades, the incidence of diabetes is expected to increase around the world (3). Complications of diabetes including cardiovascular disease, renal disease, blindness and neuropathy are more frequent and severe in the elderly (4), underscoring the need for better prevention and treatment of diabetes in this growing population.

Aging is associated with increased insulin resistance (5). Progression to diabetes is determined by the ability of the pancreatic β-cell to increase insulin production to adapt to increased insulin demand. In non-diabetic obese individuals, insulin resistance is increased but β-cell mass and insulin secretion are also increased to maintain glucose control (6), suggesting that healthy β-cells may proliferate to adapt to increased insulin demand. Autopsy studies show that β-cell mass is lost in late stage diabetes (7), highlighting β-cell regeneration as a therapeutic goal for both type 1 and type 2 diabetes patients.

Reports suggest that adaptive proliferation is reduced or entirely lost with aging in mouse and human β-cells (8–10), posing a barrier to devising novel therapies to prevent and treat diabetes through β-cell regeneration in this population. However, the mechanisms that cause β-cells to cease proliferating with age remain controversial. Some evidence suggests that aged β-cells are mostly unresponsive to signals that promote proliferation in young β-cells, indicative of a permanent cell-cycle arrest typical of senescence or terminal differentiation (10), while other studies report that proliferation can still be induced in old β-cells through stimuli such as exposure to the youthful microenvironment (11–13) or partial β-cell ablation (14). To increase relevance to the real-world population, studies on mechanisms that promote proliferation in young β-cells need to be investigated in old β-cells to test for age-dependence.

The UPR supports ER protein folding capacity critical for β-cells to produce and secrete insulin (15). UPR initiators ATF6, PERK, and IRE1 are conserved ER transmembrane proteins that communicate ER protein folding stress to other organelles [Reviewed in (16)]. We previous discovered that acute knockdown of ATF6α reduces, and overexpression increases, high glucose-induced adaptive proliferation in β-cells from mouse and human islets from a wide donor age range (17). However, whether UPR activation and UPR-dependent proliferation differed between young and old islets were not examined. Since loss of proteostasis is commonly considered a hallmark of aging (18), impaired UPR-responsive proliferation is a plausible explanation for the age-associated deficit in adaptive β-cell proliferation. Here we test the hypothesis that impaired UPR signaling is responsible for the reduction of glucose-responsive proliferation observed in aged mouse β-cells. Intriguingly, contrary to the hypothesis, we find that UPR-induced proliferation remains intact in old mouse β-cells.

## Materials and Methods

### Mice

All mouse procedures were approved by the UMass Medical School and Weill Cornell Medicine Institutional Animal Care and Use Committees. **“**Young” (10-14 weeks) and “old” (10-25 months) C57BL/6J mice of both sexes were obtained from litters bred in-house or purchased from the Jackson Laboratory. Old and young groups included a similar balance of male and female mice. Mice were housed in a 12-hour light/dark cycle with unrestricted access to chow and water.

### Islet Isolation, Dispersion, and Culture

Islets were isolated using collagenase P and density gradient centrifugation, handpicked, cultured, and dispersed in 0.05% trypsin as previously described (19). Since the islet mass of old mice is larger compared to that of young mice, for each replicate islets from 2 young mice were combined to provide sufficient material to match with each old mouse. Dispersed cells were plated in 500 µL islet complete media (ICM; RPMI containing 10% FBS (Atlanta Biologics), penicillin/streptomycin, and 5 mM glucose) on uncoated glass coverslips for immunostaining [50 islet equivalents (IEQ)] or directly onto Nunc-treated polystyrene 24-well plates (ThermoFisher) for RNA extraction (100 IEQ). 16-24 hours later the media was replaced with ICM containing 15mM glucose simultaneously with the addition of experimental treatments. Thapsigargin (Sigma-Aldrich) was added to a final concentration of 20 nM to induce moderate UPR (**Figure 3**). Ad-ATF6:DHFR (20) was added at a multiplicity of infection of 10, and cells were treated with 10 µM trimethoprim (TMP) to activate ATF6α or DMSO control at the time virus was added. All samples exposed to experimental treatments were matched with control-treated samples from the same pool of dispersed cells. Dispersed islet cells were then cultured for 72 hours. To allow quantification of S-phase entry, coverslip-containing wells were treated with 10 µg/mL Bromodeoxyuridine (BrdU; Sigma-Aldrich) for the last 24 hours.

### Immunostaining, Microscopy, and Cell Counting

Following fixation for 10 minutes in 4% paraformaldehyde and unmasking for 25 minutes in 1N HCl, immunostaining for insulin and BrdU were executed as previously described (19) using primary antibodies from Abcam (BrdU: #ab6326, insulin: #ab7842), DyLight secondary antibodies from Jackson ImmunoResearch Laboratories and DAPI (Sigma-Aldrich). Fluorescence images were acquired using a Nikon microscope. Images were blinded and the number of Insulin^+^ and BrdU^+^Insulin^+^ cells were manually counted. 3149 ± 657 β-cells were counted for the glucose dose range experiment, 1214 ± 597 β-cells were counted for the thapsigargin experiment, and 1314 ± 379 β-cells were counted for the ATF6 experiment. Data are reported as the percent of Insulin+ cells that were BrdU^+^Insulin^+^.

### Cell Lysis, RNA Extraction, cDNA Synthesis, and qPCR

Cells were washed 3x in cold PBS before lysis. Cells were lysed and kept at -80°C until RNA was extracted using the RNA/Protein Purification Plus Kit (NorgenBiotek) per the manufacturer’s instructions. 300-600 ng of total RNA was used to synthesize cDNA using the SuperScript IV VILO Master Mix (ThermoFisher) per the manufacturer’s instructions. qPCR was performed using SYBR Green and the listed primers (**Supplementary Table 1**). Relative changes in gene expression were quantified using the ΔΔCt method.

### Semi-Quantitative Gel-Based Assay for *Xbp1* Splicing

The assay was performed according to a previously published technique (21). Products were separated by electrophoresis through a 3% agarose gel, and bands were quantified using Image J.

### Statistical Analysis

Data were analyzed using GraphPad Prism 8. For all experiments, the interaction between age and the response to treatment was investigated using the Repeated Measures Two-Way ANOVA followed by Sidak’s multiple comparisons testing, or Student’s t-test. The α-level was set to 0.05. Data are presented as individual data points or mean with standard error; the number of replicates is included in each figure.

## Results

### Aging Restricts Glucose-Induced Proliferation in Mouse β-Cells

**Figure 1:**
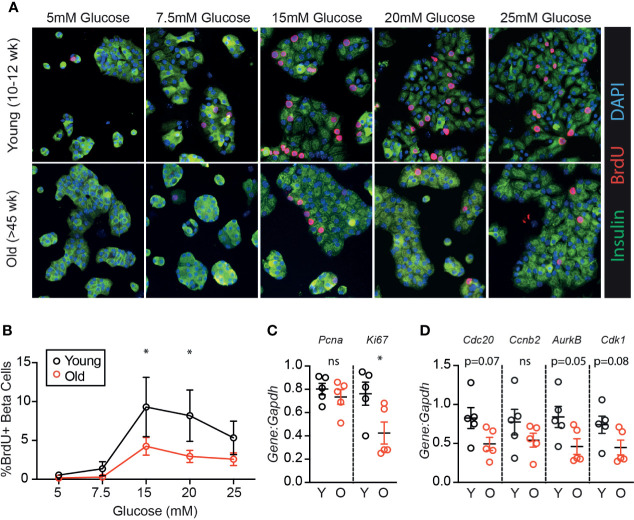
β-cells from older mice have a reduced proliferative response to high glucose compared to younger mice. Dispersed young (Y:10-12 weeks) or old (O:>45 weeks) mouse islet cells were cultured in each glucose concentration for 72 hours. BrdU was added for the final 24 hours of culture to label β-cells in S phase. **(A)** Representative immunofluorescence images of young and old dispersed mouse islet cells in each glucose concentration stained for insulin (green), BrdU (red) and DAPI (blue). Proliferating Ins^+^ β-cells are marked by BrdU^+^ nuclei. **(B)** Brdu^+^Ins^+^ cells were manually counted and represented as a % of Ins^+^ cells at each glucose concentration (n=4). Statistical analysis was performed by two-way repeated measures ANOVA and post-hoc Sidak’s multiple comparisons test. **(C, D)** qPCR of islet cells cultured in 15mM glucose quantified the relative abundance of proliferation-associated transcripts *Pcna* and *Ki67* **(C)** or *Cdc20*, *Ccnb2*, *AurkB* and *Cdk1* **(D)** in comparison to reference gene *Gapdh*. Statistical analysis was performed by *t*-test. *P < 0.05. ns, non significant.

To determine if age-associated loss of β-cell proliferation was dependent on the degree of glucose stimulus, young (10-12 weeks) and old (>45 weeks) mouse islets were trypsinized and cultured in a range of glucose concentrations (5-25 mM). Proliferation was measured by BrdU incorporation, which is a reliable marker for β-cell S-phase entry in young and old islet cells in our hands (19). Old β-cells had significantly reduced BrdU incorporation compared to young β-cells at high glucose (15-20 mM); a trend towards reduced proliferation was observed at all glucose concentrations (**Figures 1A, B**; n=4). Abundance of mRNA of two proliferation-associated markers were inconsistent; at 15 mM glucose, *Ki67* but not *Pcna* was reduced in old dispersed islets (**Figure 1C**). Proliferation markers reported to be upregulated in cycling β-cells [*Cdc20*, *Ccnb2*, *AurkB* and *Cdk1* (22)] trended towards lower expression in old dispersed islet cultures but the differences did not reach statistical significance (**Figure 1D**). Note that bulk RNA assessment has limited sensitivity to detect transcriptional differences in the small proportion of dividing β-cells in dispersed islet cultures, reinforcing the utility of immunofluorescence measurements that quantify proliferation on a per-cell basis.

### ER Stress Response Pathways Are Intact in β-Cells From Older Mice

**Figure 2:**
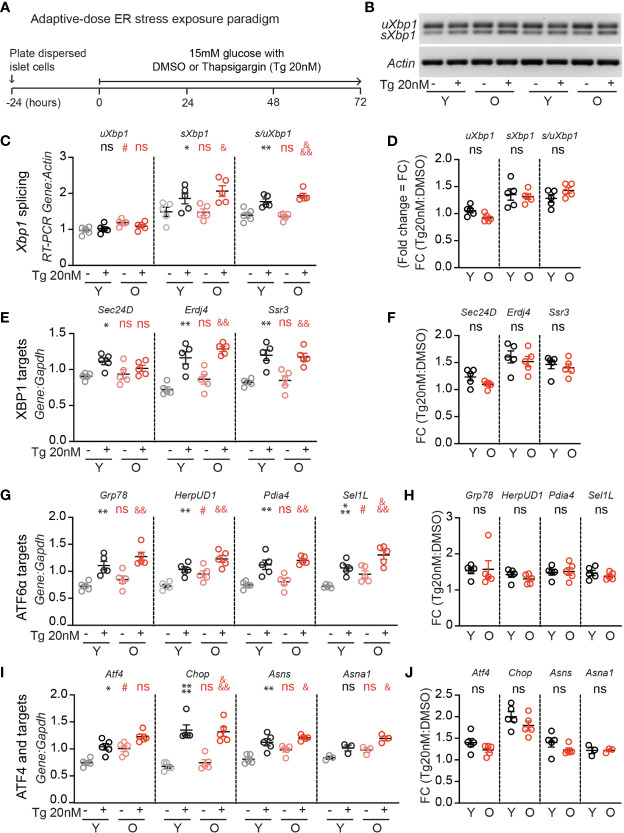
Adaptive-dose ER stressor thapsigargin induces UPR pathway activation in old islet cells similarly to young islet cells. **(A)** Timeline of thapsigargin experiments. After allowing 24 hours to recover from trypsinization in normal glucose (5mM), dispersed islets from young and old mice were cultured in 15mM glucose in the presence of low-dose thapsigargin (Tg; 20nM) or vehicle (DMSO) for 72 hours, then harvested for RNA isolation. **(B)** RT-PCR gel-based assay for *Xbp1* splicing, comparing young and old mouse islet cells cultured in high glucose with low-dose Tg or DMSO (n=5). **(C, D)** was used to quantify the relative abundance of unspliced and spliced *Xbp1* (*uXbp1* and *sXbp1*) compared to *Actin* **(C)**; fold change is shown in **(D)**. **(E, F)** qPCR assays for XBP1 targets **(E)**; fold changes are shown in **(F)**. **(G, H)** qPCR assays for Atf6 targets **(G)**; fold changes are shown in **(H)**. **(I, J)** qPCR assays for Atf4 pathway targets **(I)**; fold changes are shown in **(J)**. **(C, E, G, I)** Statistical analyses were performed by two-way repeated measures ANOVA and post-hoc Sidak’s multiple comparisons test. *Y-DMSO *vs* Y-Tg20. ^#^Y-DMSO *vs* O-DMSO. ^&^O-DMSO *vs* O-Tg20. ns, non significant. **(D, F, H, J)** Statistical analyses performed by *t*-test. One symbol, p < 0.05; two symbols, p < 0.01, three symbols, p < 0.001. ns, non significant.

Since UPR activation is required for glucose-dependent β-cell proliferation (17), and a loss of proteostasis is a hallmark of aging (18), we hypothesized that aging islets may have aberrant UPR signaling which could explain impaired glucose-responsive proliferation. To investigate, young and old dispersed mouse islet cells were cultured in high glucose media (15mM) and treated with low dose thapsigargin (20 nM) or DMSO for 72 hours, followed by RNA extraction (**Figure 2A**). Thapsigargin triggers the UPR by blocking activity of the sarco/endoplasmic reticulum Ca^2+^ ATPase (SERCA), preventing calcium uptake into the ER necessary for proper protein folding (23). *Xbp1* splicing was measured as a readout for IRE1 activity (**Figures 2B–D**) using an RT-PCR gel-based method (21), and canonical IRE1, ATF6, and PERK/ATF4 pathway targets were quantified by qPCR (**Figures 2E–J**). In 15 mM glucose, old islet cells contained slightly increased levels of unspliced *Xbp1* (**Figure 2C**), ATF6 targets *HerpUD1* and *Sel1L* (**Figure 2G**), as well as *Atf4* transcripts (**Figure 2I**), suggesting a slightly more active UPR in old, dispersed mouse islets cultured in high glucose. However, upon addition of low dose thapsigargin, young and old islets responded similarly in activating *Xbp1* splicing and expression of XBP1, ATF6 and ATF4 targets (**Figures 2B, C, E, G, I**). Aging did not alter the fold change in transcript abundance after thapsigargin treatment for any UPR marker (**Figures 2D, F, H, J**). These results suggest that loss of UPR pathway activation does not explain the age-associated reduction in glucose induced β-cell proliferation.

### Older β-Cells Retain the Proliferative Response to Low-Dose Thapsigargin

**Figure 3:**
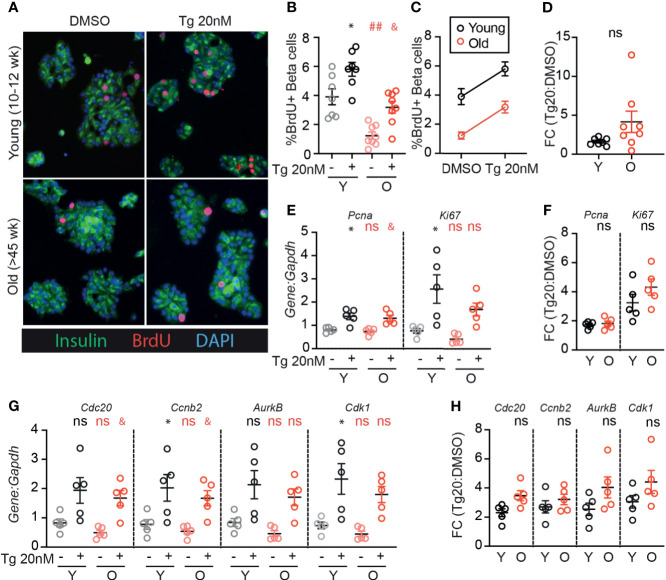
Old β-cells increase proliferation in response to adaptive-dose ER stress similarly to young β-cells. Young (Y:10-14 weeks) and old (O: >45 weeks) dispersed mouse islet cells were cultured in 15mM glucose with or without low-dose thapsigargin as described in **Figure 2**. **(A)** Representative images of young and old dispersed mouse islet cells stained for insulin (green), BrdU (red) and DAPI. **(B–D)** Brdu^+^Ins^+^ cells were represented as a % of Ins^+^ cells (n=7-8) and graphed as individual data points **(B)** or in a grouped analysis **(C)**; fold change (FC) is shown in **(D)**. **(E, F)** qPCR results for proliferation marker transcripts *Pcna* and *Ki67* **(E)**; fold changes are shown in **(F)**. **(G, H)** qPCR results for proliferation-associated transcripts *Cdc20*, *Ccnb2*, *AurkB* and *Cdk1* **(G)**; fold changes are shown in **(H)**. **(B, E, G)** Statistical analyses were performed by two-way repeated measures ANOVA and post-hoc Sidak’s multiple comparisons test. *Y-DMSO *vs* Y-Tg20 ^#^Y-DMSO *vs* O-DMSO ^&^O-DMSO *vs* O-Tg20. ns, non significant. **(D, F, H)** Statistical analyses performed by *t*-test. One symbol, p < 0.05; two symbols, p < 0.01. ns, non significant.

Although aged β-cells have impaired glucose-responsive proliferation [**Figure 1** and (24)], it is unknown whether aged β-cells can be induced to proliferate by low dose ER-stress as previously illustrated for young β-cells (17). As expected, old β-cells had reduced BrdU incorporation compared to young β-cells when cultured in 15mM glucose under DMSO control conditions (**Figures 3A–C**). However, low-dose thapsigargin significantly increased BrdU incorporation in both young and old β-cells. Although fewer old β-cells stimulated with thapsigargin had BrdU incorporation compared to young β-cells under the same conditions (**Figure 3B**), the magnitude of the pro-proliferative effect of thapsigargin was similar between young and old islet samples (**Figures 3C, D**). Proliferation-associated mRNA transcripts were increased in thapsigargin treated young and old dispersed islet cultures, and the fold change of expression upon stimulation with thapsigargin did not differ between young and old for any gene, if anything trending higher in the older samples (**Figures 3E–H**). Thus, consistent with the similar UPR pathway activation between young and old islet cells, aged mouse β-cells do not have impaired UPR-induced proliferation in this *ex vivo* culture system.

### ATF6α Activation Robustly Induces Proliferation in Older β-Cells

**Figure 4:**
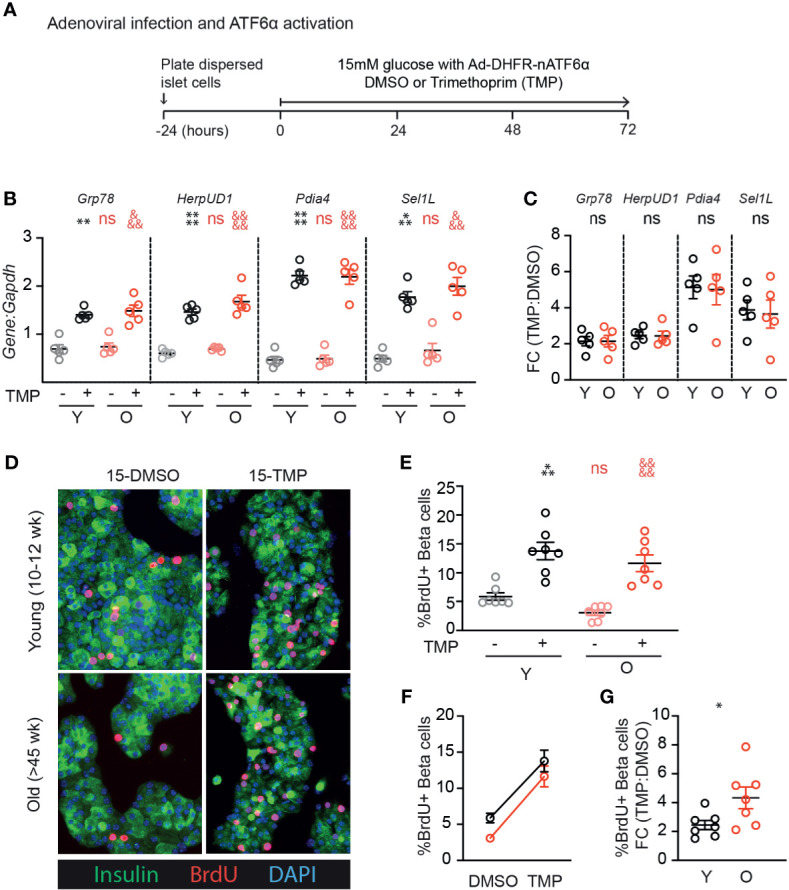
Old β-cells increase proliferation in response to ATF6α activation similarly to young β-cells. **(A)** Timeline of experiments showing Ad-DHFR-ATF6α transduction and stabilization (activation) of the DHFR-ATF6α fusion protein with trimethoprim (TMP). After 24 hours recovery, young and old dispersed mouse islet cells were transduced with Ad-DHFR-ATF6α (MOI=10) with simultaneous treatment of 10 uM trimethoprim (TMP) or vehicle (DMSO) and addition of 15mM glucose. Cultures were harvested for RNA isolation or immunofluorescence staining at 72 hours. **(B, C)** qPCR assays showed similar activation of Atf6α targets in young and old islet cells (n=5) **(B)**; fold changes are shown in **(C)**. **(D)** Representative images of young and old Ad-DHFR-ATF6α transduced islet cells stained for insulin (green), BrdU (red) and DAPI (blue). **(E, F)** %Brdu^+^ were remarkably similar between young and old Ad-DHFR-ATF6α-expressing beta cells upon activation with TMP (n=7) expressed as individual data points **(E)**, grouped analysis **(F)** and fold change **(G)**. **(B, E)** Statistical analyses were performed by two-way repeated measures ANOVA and post-hoc Sidak’s multiple comparisons test. *Y-DMSO *vs* Y-Tg20 ^#^Y-DMSO *vs* O-DMSO ^&^O-DMSO *vs* O-Tg20. **(G)** Statistical analyses were performed by *t*-test. One symbol, p < 0.05; two symbols, p < 0.01, three symbols, p < 0.001, four symbols, p < 0.0001. ns, non significant.

We previously identified ATF6α as a UPR component that is required for glucose-induced β-cell proliferation, and when overexpressed further increases proliferation in the context of 15mM glucose (17). To test whether aging β-cells have impaired responsiveness to ATF6α, we applied stress-independent activation of ATF6α using the ATF6α-dihydrofolate reductase (DHFR) fusion system previously developed by Shoulders et al. (20). Binding of the DHFR inhibitor trimethoprim (TMP) to the DHFR-domain stabilizes its folding structure; in the absence of TMP the protein remains unfolded and is rapidly degraded (20). Young and old mouse β-cells were transduced with Ad-ATF6α-DHFR and exposed to 10uM TMP or DMSO for 72 hours (**Figure 4A**). Activation of ATF6α-target genes was similar in young and old dispersed islet cells (**Figures 4B, C** **)**. Strikingly, stabilization of ATF6α with TMP caused a dramatic and significant increase in the fraction of β-cells incorporating BrdU (**Figures 4D–G**). The increased proliferation frequency of young and old β-cells upon activation of ATF6α were remarkably similar; the proliferation fold change was actually higher in the old condition (**Figures 4F, G**). These data suggest that ATF6α-driven β-cell proliferation is not lost in aging β-cells.

## Discussion

This study tested the hypothesis that reduced proliferation in high glucose in aging β-cells is due to impaired activation of UPR pathways or UPR-dependent proliferation. Contrary to the hypothesis, the results suggest that islet cells from older mice do not have impaired activation of UPR pathways in response to low dose thapsigargin. Remarkably, despite a lower proportion entering the cell cycle at baseline in 15mM glucose, old mouse β-cells also retained a robust proliferation response to mild ER stress and to ATF6 activation. These results suggest the exciting possibility that if UPR pathways can be harnessed therapeutically to increase β-cell mass, the treatment might retain efficacy in older subjects.

Age-associated loss of β-cell proliferation may be due to quiescence, terminal differentiation, or senescence (9). Although these states all feature cell cycle arrest, they are physiologically distinct. Quiescence is a normal physiological state in which cells are non-proliferative but are poised to enter the cell cycle when induced by pro-mitogenic signals. Terminal differentiation is also a normal developmental program in which an irreversible block of cell division occurs as cells acquire mature functional properties. In contrast, senescence is a pathological response to potentially oncogenic stimuli such as DNA damage (25). Senescent cells impact tissue function both by cell-intrinsic restriction of regenerative capacity as well as through paracrine effects known as the “Senescence Associated Secretory Phenotype” (SASP) (26, 27).

All three of these processes occur in β-cells and could potentially be modified by UPR activation, either through increasing likelihood that proliferation-competent β-cells enter the cell cycle, or by increasing the replication-competent pool. The β-cell quiescence period is lengthened by older age and lower ambient glucose (28). Whether UPR activation modulates the β-cell quiescence period remains unknown, but the current observations are consistent with the possibility that high glucose and UPR activation additively or synergistically shorten the quiescence period. Age-related loss of proliferation has been associated with increased insulin secretion (29–31). The short time frame of the current experiments argues against a mechanism involving prevention of terminal differentiation, but UPR activation could potentially recall β-cells from a modified terminally differentiated state. Finally, senescent β-cells are known to accumulate in the islet with age and in diabetes, and high fat diet or free fatty-acids induce senescence in the β-cells *via* p16^INK4a^ (27, 32–34). Derepression of the p16^INK4a^ locus by polycomb repressive complex 1 (PRC1) component BMI1 is another important mechanism limiting β-cell proliferation in aging (35). Although loss of p16^INK4a^ was not sufficient to restore the proliferative response to glucose in mouse islets (24), the impact of UPR activation on β-cell senescence, SASP or BMI1 remain unknown.

Intriguingly, we observed slightly increased UPR activation in old mouse β-cells at baseline in high glucose, despite lower proliferation in high glucose compared to young β-cells. This effect was more pronounced when the qPCR results were normalized to *Actin* than when normalized to *Gapdh* (data not shown), which was possibly due to a systematic reduction in *Actin* abundance in older islet cell cultures. Islet cell cultures are prepared following islet isolation and trypsinization, both of which can cause ER stress; it is unknown whether aging alters the ER stress response to these procedures. The conundrum of why older β-cells had reduced proliferation in “basal” high glucose conditions despite slightly activated UPR, whereas proliferation was robustly activated in the same cultures when further stress was applied, might be explained by time frame or intensity of stress. The low-level stress response may have been active for multiple days, bypassing a window for inducing proliferation, or it may have been of insufficient intensity or breadth to trigger cell cycle entry.

How these results relate to β-cell proliferation in the aging *in vivo* environment remains unknown. However, the diminished proliferation frequency in high glucose consistently observed in the current studies suggests that at least some portion of the *in vivo* aging lower-proliferation phenotype was replicated in these *ex vivo* cultures. Some effects of aging on β-cells are not cell-intrinsic, but rather are the result of interaction with the aged extracellular environment. For example, transplantation of old islets to young hosts restored the proliferation of aged mouse β-cells (11, 12). It is not known whether the aged microenvironment impacts UPR-induced proliferation, nor whether ATF6α activation increases β-cell proliferation *in vivo* in any mice, young or old.

This study did not assess human β-cells for loss of UPR signaling or UPR-induced proliferation. We previously reported that low dose ER stress and ATF6α overexpression activated β-cell proliferation in dispersed human islet cells, but did not compare the proliferation between young and old donors (17). In that study, islet donors ranged in age from 15-65 years; β-cells from the three oldest donors, ages 61, 63 and 65, all increased BrdU incorporation in response to low dose thapsigargin and/or tunicamycin [see (17) Supplemental Figures 13C, G, K]. Intriguingly, a single-cell RNA sequencing study reported that β-cells isolated from aged non-diabetic cynomolgus monkeys had aberrantly increased UPR expression (36). It is unknown whether UPR triggers β-cell proliferation in primate β-cells.

With respect to specific UPR pathways, this study only tested the impact of ATF6α activation on β-cell proliferation. The IRE1/XBP1 pathway also impacts β-cell proliferation, although both activation and inhibition reduced proliferation, suggesting this pathway may be less therapeutically useful as a tool to increase β-cell mass (17). Inhibition of the PERK pathway did not impact mouse β-cell proliferation (17).

Our study has weaknesses that may be addressed in future studies. We did not assess the effect of aging on the induction of proliferation and cell cycle genes across a glucose range, focusing instead on the effect of adding UPR in the context of 15mM glucose. We also did not test whether mouse sex impacts the proliferation response to thapsigargin or ATF6α activation in young or old β-cells.

In summary, the current study suggests the promising observation that older mouse β-cells retain proliferative responsiveness to UPR, and to ATF6α activation specifically. However, many important questions remain. It is unclear whether UPR-induced β-cell proliferation can be sustained over a longer duration, or how the functional state of these newly created daughter β-cells compares to the parent cells. Whether this process can be harnessed to increase β-cell mass *in vivo* in mice, or even humans, also remains untested. Future studies will be necessary to determine if modulation of ATF6α has therapeutic utility for the treatment diabetes.

## Data Availability Statement

The original contributions presented in the study are included in the article/**Supplementary Material**. Further inquiries can be directed to the corresponding authors.

## Ethics Statement

The animal study was reviewed and approved by Institutional Care and Use Committees of the UMass Medical School and the Weill Cornell Medical College.

## Author Contributions

The study was conceptualized by JS, RS, and LA. Experiments were performed and analysed by JS, CD, and RS. The first draft of the manuscript was prepared by JS (text) and RS (figures). All authors contributed to the article and approved the submitted version.

## Funding

This work was supported by NIH/NIDDK: R01DK114686 (LCA), R01DK113300 (LCA), R01DK124906 (LCA), NIH/NIGMS: R25GM113686 (JTS) and George F. and Sybil H. Fuller Foundation (LCA).

## Conflict of Interest

The authors declare that the research was conducted in the absence of any commercial or financial relationships that could be construed as a potential conflict of interest.

## Publisher’s Note

All claims expressed in this article are solely those of the authors and do not necessarily represent those of their affiliated organizations, or those of the publisher, the editors and the reviewers. Any product that may be evaluated in this article, or claim that may be made by its manufacturer, is not guaranteed or endorsed by the publisher.

## Abbreviations

ER, endoplasmic reticulum; UPR, unfolded protein response; PERK, Protein kinase R-like ER kinase; IRE1, Inositol-requiring enzyme 1; ATF4, Activating transcription factor 4; ATF6, Activating transcription factor 6; CDK, Cyclin dependent kinase.
