# Supplementary material for: Endoplasmic Reticulum Stress Induced Proliferation Remains Intact in Aging Mouse β-Cells
Source: Front Endocrinol (Lausanne). 2021 Aug 31;12:734079. doi: 10.3389/fendo.2021.734079 (PMC8438540; doi:10.3389/fendo.2021.734079)
Supplement: Supplementary file 1 [file Table_1.docx]

Supplementary Table 1

| Primers (Mouse) | Forward Primer | Reverse Primer |
| --- | --- | --- |
| *Pcna* | ACCTGCAGAGCATGGACTCG | GCAGCGGTATGTGTCGAAGC |
| *Ki67* | CTGCCTGCGAAGAGAGCATC | AGCTCCACTTCGCCTTTTGG |
| *Cdc20* | GGCACATTCGCATTTGGAACG | TAGTGGGGAGACCAGAGGATGGAG |
| *Ccnb2* | GAGAGTGAAGTCCTGGAA | GTGCTGATCTTCAGGAGT |
| *AurkB* | GCCCCTCGCGGGGAACTCTA | GGCATGCACCGACCAGCCAA |
| *Cdk1* | CAGAGATTGACCAGCTCTT | GAAAGGTGTTCTTGTAGTCC |
| *s/uXbp1 (Gel Method)* | GAACCAGGAGTTAAGAACACG | AGGCAACAGTGTCAGAGTCC |
| *Sec24D* | TGTAGGCTTTGCACCTGTTG | TCAAACCCTTCGCTGACATT |
| *Ssr3* | ACCAGAACCAGGAACAGAGTG | CCGGAAGGAGAAAGACGAA |
| *Erdj4* | TGAATTTGCAGAGGTTTCACTG | CAAACTCAGCCCGACACATA |
| *Grp78* | AGGACAAGAAGGAGGATGTGGG | ACCGAAGGGTCATTCCAAGTG |
| *HerpUD1* | CAACAGCAGCTTCCCAGAAT | CCGCAGTTGGAGTGTGAGT |
| *Hyou1* | TAGCCAGGTGTTCTCGAAGC | GACTAAGGAGGCTGGGATGC |
| *Pdia4* | ATCGCCAAGATGGGATGCTAC | CTTGGTCCTGCTCCTCTTTG |
| *Sel1L* | GTCGTCTTTTGGCAGCATCT | GATCTCCGAATCCAAGCAG |
| *Atf4* | GGACAGATTGGATGTTGGAGAAAATG | GGAGATGGCCAATTGGGTTCAC |
| *Chop* | CACATCCCAAAGCCCTCG | CTCAGTCCCCTCCTCAGC |
| *Asns* | TGACCCGCTGTTTGGAATG | CTGTAGCGCCTTGTGGTTGTA |
| *Asna1* | CGCTGGAACCCACGCTTAG | CAGGTGGTCTTACCAACGCC |
| *Gapdh* | AGGTCGGTGTGAACGGATTTG | TGTAGACCATGTAGTTGAGGTCA |
| *Actin* | AGCCATGTACGTAGCCATCC | CTCTCAGCTGTGGTGGTGAA |
